# Intermediate Cells of Dual Embryonic Origin Follow a Basal to Apical Gradient of Ingression Into the Lateral Wall of the Cochlea

**DOI:** 10.3389/fcell.2022.867153

**Published:** 2022-03-08

**Authors:** Justine M. Renauld, Vibhuti Khan, Martín L. Basch

**Affiliations:** ^1^ Department of Otolaryngology, Head and Neck Surgery, Case Western Reserve University School of Medicine, Cleveland, OH, United States; ^2^ Department of Genetics and Genome Sciences, Case Western Reserve School of Medicine, Cleveland, OH, United States; ^3^ Department of Biology, Case Western Reserve University, Cleveland, OH, United States; ^4^ Department of Otolaryngology, Head and Neck Surgery, University Hospitals, Cleveland, OH, United States

**Keywords:** intermediate cells, neural crest, stria vascularis, inner ear, cochlea, schwann cell precursor, melanoblast, inner ear development

## Abstract

Intermediate cells of the stria vascularis are neural crest derived melanocytes. They are essential for the establishment of the endocochlear potential in the inner ear, which allows mechanosensory hair cells to transduce sound into nerve impulses. Despite their importance for normal hearing, how these cells develop and migrate to their position in the lateral wall of the cochlea has not been studied. We find that as early as E10.5 some Schwann cell precursors in the VIIIth ganglion begin to express melanocyte specific markers while neural crest derived melanoblasts migrate into the otic vesicle. Intermediate cells of both melanoblast and Schwann cell precursor origin ingress into the lateral wall of the cochlea starting at around E15.5 following a basal to apical gradient during embryonic development, and continue to proliferate postnatally.

## Introduction

The stria vascularis is a specialized epithelium that actively pumps potassium into the scala media to generate the positive endocochlear potential essential for sensory hair cells to transduce sound vibrations into action potentials (Hudspeth, 1989; Steel and Barkway, 1989; Takeuchi et al., 2000; Hibino et al., 2010). This specialized epithelium located on the lateral wall of the cochlea is composed of three different cell types: marginal cells, intermediate cells, and basal cells, each with a distinct embryonic origin. Marginal cells are derived from the otic epithelium and line the lumen of the scala media (Sagara et al., 1995). Intermediate cells are melanocyte-like cells derived from the neural crest (Cable et al., 1992). Basal cells are adjacent to the spiral ligament and are derived from otic mesenchyme (Trowe et al., 2011). Mesodermally-derived blood vessels are interspersed throughout the stria vascularis in close association with intermediate cells. (Steel and Barkway, 1989). In the vestibular organs, except for the sacculus, the potassium transport is mediated by dark cells. These dark cells express the same channels as marginal cells of the stria vascularis and are in close associations with neural crest derived pigmented cells underneath that are thought to regulate calcium homeostasis (Ciuman, 2009).

The presence of intermediate cells in the inner ear has been shown to be essential for hearing and developmental defects affecting intermediate cells are responsible for several congenital deafness (Edery et al., 1996; Matsushima et al., 2002; Ni et al., 2013). Those developmental disorders are part of a larger group of diseases called neurocristopathies and originate from defects in development, differentiation or survival of neural crest cells (Ritter and Martin, 2019). Like most pigmented cells of vertebrates, intermediate cells originate from neural crest cells.

The neural crest is a transient population of multipotent embryonic vertebrate cells. It gives rise to multiple derivatives including most of the pigmented cells in the body, the peripheral nervous system and most of the craniofacial skeleton [Reviewed in (Basch et al., 2004; Sauka-Spengler and Bronner-Fraser, 2008)]. In avian embryos, most neural crest derivatives are originated from an early ventral migrating population of neural crest cells, while melanocytes are originated from a later migrating subset of neural crest cells that migrate dorsolaterally between the dermomyotome and the skin (Erickson and Goins, 1995; Kuo and Erickson, 2010). Recent studies in mouse have shown that in addition to neural crest that take the dorsolateral pathway, a significant number of melanocytes are derived from the re-differentiation of nerve-associated Schwann cell precursors that came from ventromedial migrating neural crest cells (Adameyko et al., 2009; Adameyko et al., 2012; Yoshimura et al., 2013; Furlan and Adameyko, 2018).

Despite being essential for hearing, little is known about the development of the stria vascularis. In this paper, we focused on the development of the pigmented cells of the stria. We followed their development all the way until their differentiation as intermediate cells. We analyzed the timing of their formation, their incorporation into the lateral wall of the cochlea, their differentiation, and their proliferation state. We found that intermediate cells can originate directly from dorsolateral migrating melanoblasts or from the differentiation of Schwann cell precursors. They incorporate into the lateral wall of the cochlea starting at around E15.5 following a basal to apical gradient reminiscent of the differentiation of hair cells in the organ of Corti. Intermediate cells continue to proliferate from embryonic stages until maturity in the postnatal strial epithelium.

## Materials and Methods

### Experimental Animals

For this study we used the following mice: Wnt1Cre2 (stock no. 022137), tamoxifen inducible Wnt1CreER (stock no. 008851), Ai3-YFP [stock no.007903: ROSA26 enhanced yellow fluorescent protein (EYFP) reporter], Ai9-TdTomato (stock no. 007909), and Plp1-EYFP (stock no. 033357). All mice were obtained from Jackson Laboratory and bred in the animal facility of Case Western Reserve University following the IACUC guidelines for the use and care of laboratory animals (protocol no. 2018-0034). We set timed matings between heterozygous Wnt1Cre, Wnt1creER males with homozygous Ai3-YFP or Ai9-TdTomato reporter females. The following morning, vaginal plug visualization was considered embryonic day 0.5 (E0.5) and the day mice were born was considered as postnatal day 0 (P0). We analyzed at least three littermates and two litters from each embryonic and postnatal stage studied.

### Lineage Tracing and Tamoxifen Administration

To fate map the contribution of neural crest cells to the inner ear we crossed hemizygous Wnt1Cre males with homozygous Ai3-YFP or Ai9-TdTomato reporter females and harvested embryos from E8.5 to E17.5 and dissected cochleae from pups at postnatal days P0, P6 and P14. To dissect the temporal contributions of neural crest, we crossed tamoxifen inducible Wnt1CreERT2 hemizygous males to Ai3-YFP homozygous females. To induce the activity of the CRE recombinase we administered a single dose of 2 mg tamoxifen with 2 mg progesterone (20 mg/ml in peanut oil) via oral gavage to the pregnant females at E8.5, E9.5, E10.5 and E11.5 and collected embryos at P0. To count the number of intermediate cells in stria vascularis we have analyzed three pups from three different pregnant females for each stage. We counted nine sections of 12 µm thickness covering 108 µm of the cochlea length from each of the three pups for a total of 27 sections per stage. Statistical analysis was done by using a 2-tailed student t test. A *p*-value ≤0.05 was considered as statistically significant.

### Genotyping

Genotyping of embryos and pups was determined by PCR and confirmed by the presence of EYFP fluorescence under a Mercury lamp. Primers used for CRE genotyping were CreF: GCC​TGC​ATT​ACC​GGT​CGA​TGC​AAC​GA and CreR: GTG​GCA​GAT​GGC​GCG​GCA​ACA​CCA​TT.

### Edu (5-Ethynyl-2-deoxyuridine) Labeling and Cell Death

To analyze the cell proliferation in stria at embryonic day E16.5 and E18.5, a single dose of EdU (100 µL/10 g of body weight, C10339, Invitrogen) was administered to timed pregnant females. The cochleae of the neonatal pups were dissected at P0. For the postnatal cell proliferation analyses a single dose of EdU (250 µg/10 g of body weight) was administered subcutaneously to the pups at P0, P3, P6 and P14. These pups were euthanized 6 h after the administration of EdU and their cochleae were extracted. EdU detection was performed according to the manufacturer’s instructions with a ClickiT EdU Alexa Fluor 594 Imaging kit (C10339, Invitrogen). In Edu proliferation assay we have administered Edu in two different pregnant females or two different litters for each stage. For each stage analyzed, we have used three pups (nine sections from each pup). A total of 27 sections each of thickness 12 µm (covering around 108 µm of the cochlea length in each sample) were analyzed. Statistical analysis was done by using a 2-tailed student t test. A *p*-value ≤0.05 was considered as statistically significant.

### Histology

Pregnant females were euthanized in a CO2 chamber for at least 10 min and cervical dislocation was used as a secondary method, embryos between E8.5 and E17.5 were collected. P0, P3, P6 and P14 pups were placed in a CO_2_ chamber for over 30 min and immediately afterwards decapitated. Inner ears from P3, P6 and P14 pups were dissected prior to fixation. Embryos at E8.5, E9.5 and E10.5 were fixed with 4% paraformaldehyde for 1 hour at room temperature. All other tissues were fixed in 4% paraformaldehyde overnight at 4°C. After fixation all the samples were washed three times for 10 min in PBS. Inner ears from pups older than P3 were transferred to a 0.5M EDTA solution at 4°C for the decalcification. EDTA was changed daily until decalcification was complete (determined by gently pushing on the tissue with forceps). Thereafter all the samples were transferred to 30% sucrose in PBS at 4°C until fully sink. The embryos and the dissected inner ears were embedded in plastic cryomolds filled with OCT or gelatin-sucrose (7.5–15%) in a proper orientation and frozen either in dry ice or liquid nitrogen (embryos; P0, P3, P6 and P14 respectively). The inner ears (P0, P3, P6 and P14) were cut in 12 µm thick sections for immunohistochemistry and EdU cell counting. E8.5-E105 embryos were cut in 10 µm thick sections in three different planes (coronal, transverse and sagittal) for immunohistochemistry. E11.5 to E17.5 embryos were cut in 12 µm thick sections in a transverse plane for immunohistochemistry.

### Immunohistochemistry

The cryosections were defrosted and washed with 1 × PBS for 5 min. The tissue permeabilization was done washing in 1% Triton X-100 followed by three washing in PBS for 5 min. Prior to immunostaining antigen retrieval was done by boiling the sections for 10 min in antigen retrieval (H-3300, Vector Laboratories). Thereafter, sections were blocked for 30 min using 10% lamb serum in PBS/0.1% Triton × 100 or PGT [PBS-gelatin (0.25%)-triton (0.3%)]. Sections were incubated in the primary antibodies overnight at 4°C. The next day, sections were washed three times in PBS for 10 min and then incubated in the corresponding secondary antibody for 1 h at room temperature. Sections were washed three times with PBS for 10 min and then mounted using DAPI Fluoromount (Southern Biotech).

Primary antibodies used in this study were anti-GFP (Abcam 13,970, chicken, 1:1,000), Caspase 3 (Cell signaling technology 9,662, rabbit, 1:1,000), CD44(Thermo Fisher Scientific MA4405, rat, 1:250), connexin 26(Alonome labs ACC-212, rabbit, 1:100 ), DCT (Abcam 74,073, rabbit, 1:100), KCNQ1 (Santa Cruz sc-20816, rabbit, 1:250), MITF (R&D Systems AF5769, goat, 1:500), P75 (Advanced Targeting Systems AB-N01, rabbit, 1:500), Prox1 (rabbit, 1:750). Secondary antibodies used were Alexa Fluor 488 goat anti-chicken IgY (ThermoFisher Scientific A11039, 1:1,000), Alexa Fluor 568 goat anti-rabbit IgG (ThermoFisher Scientific A11036, 1:500), Alexa Fluor 633 goat anti-rabbit IgG (ThermoFisher Scientific A21070, 1:500), anti-goat 594 (Jackson Immunoresearch, 705-005-003, 1:300).

## Results

### Neural Crest Migration Into the Inner Ear

**FIGURE 1:**
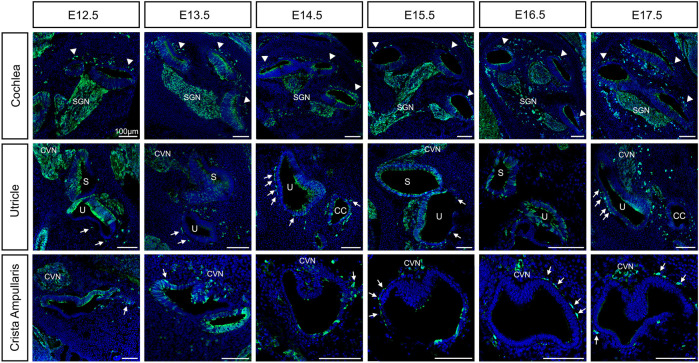
Neural crest contributions to the inner ear during embryonic development from E12.5 to E17.5. Lineage tracing of neural crest cells using Wnt1-Cre2 mice crossed to an EYFP reporter line was visualized by staining with the anti-GFP antibody in green and DAPI for nucleus in blue. Arrowheads: intermediate cells of the stria vascularis; Arrows: melanoblast of the vestibule; CVN: Cochleo-vestibular neurons; SGN: Spiral ganglion neurons; S: saccule; U: utricle. Scale bars: 100 µm.

To understand the precise development of intermediate cells in the cochlea, we took advantage of genetic lineage tracing using a Wnt1-Cre2 crossed to Ai3-YFP mouse reporter line to label neural crest cells just before they undergo their epithelial-to-mesenchymal transition and follow their development in the inner ear (Figure 1). This combination of mouse lines has been commonly used to label neural crest cell derivatives in mice (Danielian et al., 1998; Chai et al., 2000; Freyer et al., 2011). In the mouse, neural crest cells begin to migrate around the time of neural tube closure at around E8.5 (Serbedzija et al., 1992; Trainor, 2005).

As early as E10.5, we can detect MITF positive cells, one of the earliest melanoblast markers, both in the delaminating ganglion and migrating into the otic vesicle from the dorsal neural tube (Supplementary Figure S1). E11.5 we continue to see MITF positive cells in the periphery of the VIIIth ganglion and in the proximity of the inner ear (Figure 4, left panel).

By E12.5, neural crest cells that form glial cells are surrounding the ganglion. In addition, we observe neural crest cells migrating and lining up just outside the roof of the cochlear duct (arrowheads, Figure 1). In the vestibular organs, neural crest cells are migrating to and surrounding the utricle and the saccule and are already lining the ampullae of the semicircular canals where they’ll form close associations with the dark cells (arrows, Figure 1). As the cochlea elongates we observe more neural crest cells lining outside the cochlear duct at the site of the future lateral wall, up until E14.5. In the vestibular organs, cells have reached their final positions in the utricle and they begin to localize under the crista of the ampullae in the semicircular canals (arrows, Figure 1). Interestingly, we observed Wnt1Cre2-YFP positive cells scattered throughout the epithelium of the cochlea (see discussion). We also quantified the proportion of hair cells and supporting cells carrying the YFP expression at P0 (Supplementary Figure S2B).

### Ingression of Intermediate Cells in the Developing Stria Vascularis Follows a Base-to-Apical Gradient and Precedes Morphological Maturation

**FIGURE 2:**
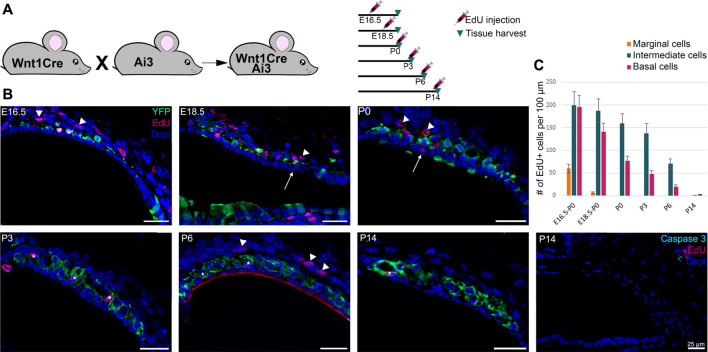
Intermediate cells in the developing stria vascularis follow a base-to-apical gradient of ingression and undergo morphological maturation at early postnatal days. **(A)** Immunostaining of E16.5 Wnt1cre-YFP mouse with white dashed square magnified in insets. Future intermediate cells migrate (arrows) then line up on top of the cochlear duct and ingress in the lateral wall of the cochlea following a basal to apical gradient. CD44 (in red) is a cell membrane marker of melanoblast that shows the protrusion in the marginal cells layer (Arrowheads). 1: apex, 2: mid apex, 3: mid base, 4: base. **(B)** Schematic representation of the base to apex semi-ingression process. Melanoblast are represented in dark pink at the surface of the lateral wall. **(C).** Immunostaining of P0, P6 and P32 Wnt1Cre-YFP mouse with white dashed square magnified in insets. Green cells in the stria vascularis are intermediate cells labeled by Wnt1-CRE2 crossed to a EYFP reporter. KCNQ1 and Connexin 26, markers of marginal and basal cells respectively, are both in purple to delimit the intermediate cell region in the stria vascularis.

**FIGURE 3:**
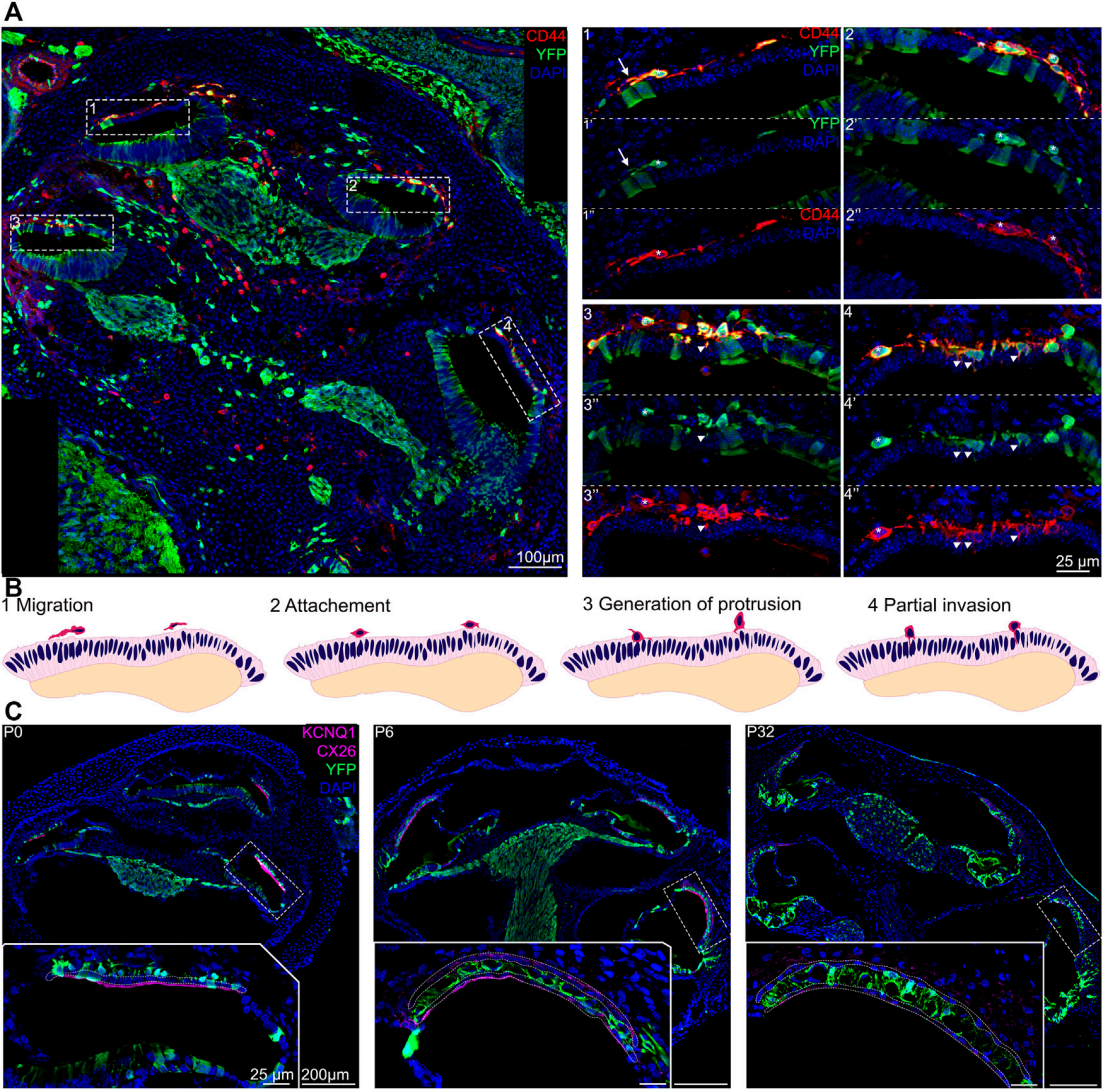
Proliferation in the stria vascularis decreases 2 weeks after birth. **(A)** Schematic of the experimental using Edu injection at different time points during development. **(B)** Immunostaining of Wnt1Cre-YFP mice from P0 to P14 with EdU labeling (dark pink) and DAPI, a nuclear marker in blue. Some Edu positive marginal, intermediate and basal cells are shown by arrows, stars and arrowheads respectively. Anti-activated caspase 3 labeling (in turquoise) shows no significant cell death in the stria vascularis during development (right panel). **(C)** Graphic of the average proliferation in the three different layers of the inner ear from E16.5 to P14. For each stage *n* = 3.

**FIGURE 4:**
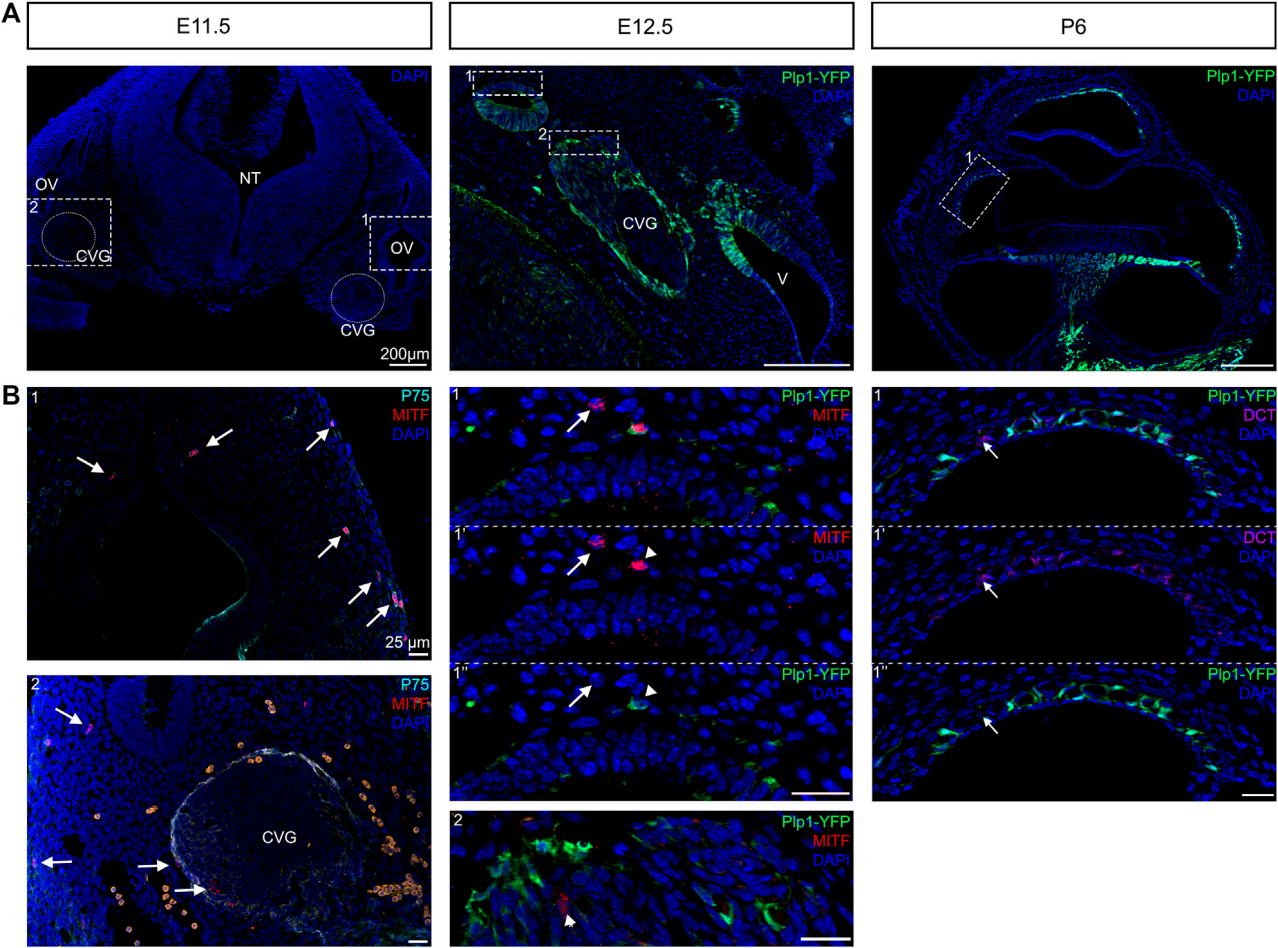
Dual embryonic origin of intermediate cells. Immunostaining for melanoblast with MITF labelling (in red) and neural crest cells with P75 (in turquoise) shows at E11.5 the presence of melanoblast between the dermis and the otic vesicle and between the otic vesicle and the neural tube (Arrows). Lineage tracing of Schwann cell precursors using Plp1-YFP reporter line at E12.5 and P6. Immunostaining for melanoblast with the MITF antibody (red) and DAPI for nucleus (blue) show cells with both markers (arrowheads) or only melanoblast marker (arrows). Most intermediate cells express the glial cell marker PLP (green) and melanocyte markers DCT (in purple) at P6 except one cell that only express DCT (arrow). CVG: cochleaovestibular ganglion, NT: neural tube, OV: otic vesicle, V: vestibule.

Around E15.5 cells that were lining the roof of the basal turn of the cochlea begin to extend protrusions into the single cell epithelium that forms the roof of the cochlea at this stage. This partial invasion continues until birth and follows a basal to apical gradient reminiscent of the gradient of differentiation in the organ of Corti. At E16.5, the different steps of development can be all seen at the different positions in the same cochlea section as shown in Figure 2A. CD44, a cell surface adhesion receptor which labels intermediate cells (Morris et al., 2006; Rohacek et al., 2017), is shown in red to visualize the cell membrane of melanocytic cells during the ingression process (Figure 2A, right panel). At this stage, we can see the future intermediate cells migrating above the marginal cells layer at the apex, characterized by an elongated shape (arrows, Figure 2A–1). At the mid-apex level, the future intermediate cells present a round shape as they cease to migrate and begin to attach to the marginal cells layer (Figure 2A–2). The stage is followed by an increase in protrusions toward the marginal cell layer as seen in the mid-base level (arrowheads, Figure 2A–3). At the base, almost all intermediate cells started to partially invade the marginal cell layer (Arrowhead, Figure 2A–4). The ingression of the intermediate cells into the stria vascularis is represented in a schematic (Figure 2B). As development proceeds, intermediate cells at the basal turns are already positioned between marginal and basal cells that are coalescing on the lateral wall of the cochlea (Figure 1 E17.5, Figures 2A–4,C).

At P0, the future intermediate cells have completed their migration into the lateral wall of the cochlea between the marginal cell layer and the basal cells layers. At this stage, the cells have not fully differentiated and they exhibit markers of both neural crest cells and intermediate cells (Renauld et al., 2021). Neonatal intermediate cells are round in shape and individual cells are seen scattered throughout the stria between marginal and intermediate cells, from the spiral prominence to Reisner’s membrane. By P6, the stria is thicker, intermediate cells begin to adopt a more columnar shape in opposition to marginal cells that become a thinner layer one cell thick. At this stage, intermediate cells begin to form close association with blood vessels that have invaded the stria from a capillary plexus in the spiral ligament (Figure 2C and (Ando and Takeuchi, 1998). As early as P14 the cells are fully matured, they form extensive interdigitations with both marginal and basal cells and they occupy the entire width of the stria from the outer sulcus to Reisner’s membrane [Figure 2C; (Cable et al., 1992)].

### Intermediate Cells Proliferate Until Maturation of the Stria Vascularis

During the development of the inner ear, to accompany the growth of the cochlea, the stria vascularis increases dramatically in width and length. The extension of the stria could be achieved by cell movement and growth, cell proliferation, or a combination of both. To address these possibilities, we analyzed cell proliferation in the stria starting at embryonic day E16.5 through P14 by administration of EdU, a thymidine analogue, to track newly synthesized DNA reflecting dividing cells. For embryonic stages, one pulse of EdU was administered to the pregnant female and the embryos were collected at P0. For postnatal stages, we analyzed EdU incorporation 6 h after subcutaneous administration. The design of the experiment is summarized in Figure 3A. We observed a rapid decline in cell proliferation in the marginal cells at late embryonic stages and no proliferation after P0. Intermediate and basal cells continue to proliferate after birth, but the rate of proliferation decreases with development and we observed little proliferation after P6 (Figure 3B). The quantification is shown in Figure 3C. Our results suggest that growth of the stria is driven by extension of intermediate cells likely due to a combination of cell movement and cell proliferation. In addition to cell proliferation, we analyzed cell death in the stria using an antibody against activated caspase-3. Although we did not see any significant cell death in any of the cell layers of the stria, occasionally we observed some caspase-3 positive cells within the fibroblasts of the spiral ligament (Figure 3C and Data not shown).

### Dual Embryonic Origin of Intermediate Cells

Recent findings have shown that there is contribution of Schwann cell precursors on the peripheral nerves to extracutaneous melanocytes such as the melanocytes of the inner ear (Bonnamour et al., 2021; Kaucka et al., 2021). Bonnamour and colleagues used a Dhh-Cre line to genetically trace Schwann cell precursors in the inner ear and found that up to 90% of the intermediate cells of the stria vascularis were labeled by Dhh tracing. Kaucka used plp1-CreERT2 to show that the contribution of Schwann cell precursors in the inner ear melanocytes decreased after E15.5. To confirm these recent findings and address the timing of contribution of nerve-associated Schwann cell precursors to intermediate cells of the inner ear, we used genetic lineage tracing to label Schwann cell precursors together with melanoblasts markers. A Plp1-YFP mouse strain was used to label Schwann cell precursors at E12.5. Plp1 has already been studied extensively between E11.5 and E15.5 for Schwann cell lineage tracing (Adameyko et al., 2009; Adameyko et al., 2012). As early as E11.5, we observed MITF positive cells between the dermis and the otic vesicle, originating from the dorsolateral migration pathway of neural crest (arrows, Figure 4B1). In addition, we observed MITF positive cells in the cochleo-vestibular ganglion, suggesting that melanocytes in the inner ear can originate from the cochleovestibular nerve via Schwann cells precursors adopting a melanocytic fate (arrows, Figure 4B2, E11.5). At E12.5, melanoblasts originating from the dorsolateral pathway (Plp1 negative, arrow) or from Schwann cell precursors (Plp1 positive, arrowhead, asterisk) are both visible in the future lateral wall of the cochlear duct (Figure 4B). Interestingly, in our mix-background mice, PLP protein is still expressed in the vast majority of strial intermediate cells at postnatal day 6 (Figure 4C, right panel). However, some DCT positive intermediate cells are Plp1 negative (Figure 4C, arrow), suggesting that while the majority of intermediate cells originate from Schwann cell precursors, there are some that come from direct melanoblast migration. PLP expression could allow us to differentiate the two populations of intermediate cells in the mature stria vascularis.

### Early Migratory Neural Crest Cells Give Rise to Both Glia and Melanocytes in the Inner Ear

Previous research has shown that in the trunk, avian and rodent neural crest cells behave differently with an early ventral pathway followed by a delayed dorsolateral migration of neural crest cells in the chick in contrast to a simultaneous migration in the mouse (Kuo and Erickson, 2010; Kuo and Erickson, 2011). In opposition to the trunk neural crest cells, the cephalic neural crest cells delaminate before the neural tube closure in a continuous wave and is believed to show little variation between species (Theveneau and Mayor, 2012). However, the time window of delamination of future melanocytes has been less studied in the cephalic location.

**FIGURE 5:**
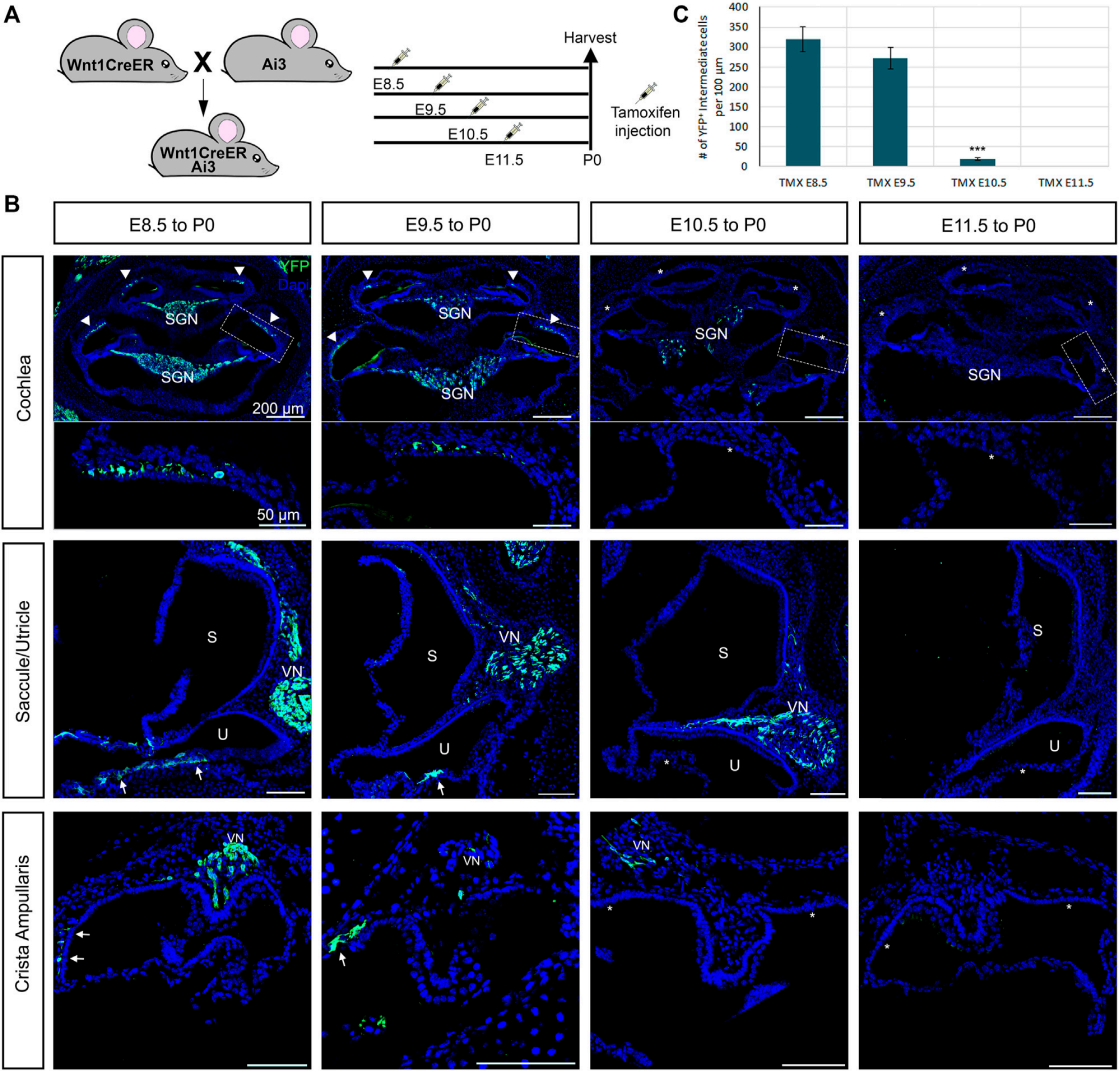
Early migratory neural crest cells give rise to both glial cells and melanocytes cells in the inner ear. Inducible Wnt1-CreER shows no late-migrating neural crest contribution to the inner ear. **(A)** Tamoxifen was administered to pregnant females via oral gavage at the ages indicated in the experimental design and collected and analyzed at P0. **(B)** Immunostaining of Wnt1CreER-YFP mice following the experimental design at P0 with YFP (green) and DAPI for nuclear labelling. Arrows indicate the melanoblast of the vestibules, the arrowheads the intermediate cells of the stria vascularis and the stars the missing labelling in the melanoblasts and intermediate cells. **(C)** Graph shows the average number of labeled intermediate cells after administration of tamoxifen at each stage. *n* = 3. CVN: Cochleo-vestibular neurons; VN: vestibular neurons; SGN: Spiral ganglion neurons; S: saccule; U: utricle.

To determine whether glial cells and melanocytes in the mouse inner ear originate from distinct temporal subpopulations of neural crest, we used an inducible Wnt1-Cre (Wnt1-CreERrT2) line crossed to a YFP reporter to label early- and late-migrating populations of neural crest. Our results are summarized in Figure 5. We administered tamoxifen *via* oral gavage to pregnant females at E8.5, E9.5, E10.5 and E11.5. and analyzed YFP expression at P0, the experimental design is represented in Figure 5A.

If there was a temporal difference in the early vs. late subpopulations of migrating cephalic neural crest, as in avian trunk embryos, we would expect labeling only intermediate cells when tamoxifen was administered at E10.5 but not glial cells. Administration of tamoxifen at E8.5 recapitulates the non-inducible Wnt1-Cre2, labeling all neural crest derivatives in the inner ear (Arrowheads, Figure 5B). We observed a similar result when we administered tamoxifen at E9.5 although slightly fewer intermediate and glial cells were labeled when compared to tamoxifen administration at E8.5. Surprisingly, when we administered tamoxifen at E10.5, most samples presented only a few glial cells labelled and no intermediate cells or melanoblasts from the vestibule (Figure 5B). We speculate that this result could be due to a lower number of melanoblasts in comparison to glial cells in the cochlea. Alternatively, melanoblasts from the inner ear stop delaminating from the neural tube at an earlier stage than glial cells, or Schwann cell precursors complete their differentiation into melanocytes before E10.5. We did not observe any labeled intermediate or glial cells after giving tamoxifen at E11.5 (Figure 5B). The quantification of the labelled intermediate cells is represented in Figure 5C.

Similar to what we saw in the cochlea, in the vestibular organs we observed a decreased number of labeled cells as we increased the stage of tamoxifen administration (Arrows, Figure 5B).

## Discussion

Neural crest cells give rise to a wide variety of derivatives in vertebrates, including craniofacial bones, peripheral nervous system, and pigmented cells in the body. In the inner ear, the contribution of neural crest to the lateral wall of the cochlea and the glia of the spiral ganglion have been described (Kuo and Erickson, 2010; Freyer et al., 2011; Sandell et al., 2014). The cochleovestibular ganglion, which innervates the cochlea and the vestibular organs, is composed of neurons derived from the otic placode (Rosenbluth, 1962; D’Amico-Martel and Noden, 1983; Breuskin et al., 2010) and glial cells derived from the neural crest (D’Amico-Martel and Noden, 1983). Intermediate cells of the stria vascularis are neural crest derived melanocytes, described by Steel et al. (Steel and Barkway, 1989). Although these neural crest contributions in the inner ear have been known for some time, the timing of how these cells migrate and how they differentiate in their proper locations during development was not known. In addition, new data has revealed that pigmented cells in the cochlea can originate from Schwann cell precursors (Bonnamour et al., 2021; Kaucka et al., 2021). In this study we characterized the temporal migration of melanoblasts into the inner ear, their dual embryonic origin, their proliferation, morphological maturation, and the subsequent postnatal differentiation into intermediate cells.

### Early Migration of Neural Crest

The neurons of the cochleovestibular ganglion delaminate from the anteroventral portion of the otic vesicle and aggregate (Altman and Bayer, 1982; Carney and Silver, 1983). As the ganglion is forming, Schwann cell precursors coalesce around the developing neurons to form the glial cells. These Schwann cell precursors are neural crest derived. It had been proposed that streams of neural crest cells migrate from the hindbrain at the level of the fourth rhombomere directly into the developing ganglion at around E10.5 and envelop the neurons of the cochleovestibular nerve (Sandell et al., 2014). At this stage, we have already seen MITF positive cells migrating into the otic vesicle from the dorsolateral pathway, as well as MITF positive cells in the forming ganglion (Figure 4). MITF is an early critical transcription factor that drives melanoblast specification (Goding, 2000). MITF expression in a number of Schwann cell precursors in the ganglion is consistent with the idea of these cells adopting a melanoblast fate before migrating into the otic vesicle. Our observations are consistent with reports from Kaucka et al., who in addition to MITF positive cells migrating both from the neural crest and from the VIIIth ganglion, also described melanocytic cells migrating into the otic vesicle posteriorly from the IX and X cranial nerves (Kaucka et al., 2021). As the cochlea begins to elongate, Schwann cell precursors keep coalescing around the neurons of the VIIIth to form glia, and some differentiate into melanocytes. MITF positive cells migrate to the roof of the cochlea in the site of the future lateral wall. By E12.5 and until E14.5 we see these cells lining up just outside of the cochlea, possibly waiting for a signal to incorporate into the stria vascularis.

### Intermediate Cells Follow a Basal to Apical Gradient of Ingression Into the Lateral Wall of the Cochlea

Shibata et al. described the incorporation of neural crest cells into the lateral wall as a three-step process (Shibata et al., 2016). First, cells migrate to the location of the future stria vascularis in response to yet unknown signals. We observe this from the formation of the cochlear duct onwards. Second, the cells attach to the site where they will incorporate. At around E14.5, we see cells that are going to ingress in the lateral wall of the cochlea begin to line up just outside the site of the future stria vascularis in the cochlear duct. Third, the melanocytes incorporate into the cochlear epithelium. We observe this ingression of cells into the lateral wall beginning at around E15.5 at the base of the cochlea. The ingression seems to follow a basal to apical gradient reminiscent of the gradient of differentiation in the organ of Corti and the formation of the scala vestibuli and scala tympanii (Figures 2A,B and (Chen et al., 2002)). Locher et al. published a basal to apical gradient of epithelial invasion in human cochlea at W14- W16 (Locher et al., 2015). As development proceeds, cells continue to ingress in the lateral wall at the midturn of the cochlea and finally at the apex prior to birth. Interestingly, Hgf and EdnrB, are both expressed by migratory neural crest cells that will become intermediate cells. Both these genes also exhibit a basal to apical expression pattern in intermediate cells that support our observation. However, without the lineage tracing, we could not rule out the possibility that expression of these genes was turned on in a graded manner as opposed to cells ingressing following the gradient (Shibata et al., 2016; Renauld et al., 2021). These results suggest that there might be a graded signal or signals that instruct the neural crest cells when to begin ingressing into the lateral wall. There is evidence to suggest that one such signal could be hepatocyte growth factor Hgf via the receptor c-Met, whether this signaling is directly or solely responsible for the gradient of incorporation of intermediate cells into the cochlea needs further study (Shibata et al., 2016; Ohyama, 2017; Morell et al., 2020).

### Intermediate Cells of the Cochlea Likely Originate From Schwann Cell Precursors

The traditional view of melanocyte formation is that melanocytes arise directly from a late-migrating population of neural crest that migrate through a dorsolateral pathway under the dermis and populates the skin. The early-migrating neural crest follow a medio ventral path of migration and form the dorsal root ganglia and eventually neurons and glia of the peripheral nervous system and the rest of the neural crest derivatives. These ideas were supported by quail-chick grafts and vital dye lineage tracing experiments in the chick embryos as well as genetic targeting experiments in the mouse (Le Douarin and Teillet, 1973; Erickson et al., 1992; Serbedzija et al., 1992; Erickson and Goins, 1995). However, recent experiments have shown that both trunk and cranial melanocytes have two distinct cellular origins: from melanoblast precursors directly derived from migrating neural crest and from nerve-associated Schwann Cell Precursors that also give rise to glial cells and are derived from the early-migrating population of neural crest (Adameyko et al., 2009; Adameyko et al., 2012; Bonnamour et al., 2021). Since then several reports have confirmed this dual origin of melanocytes. Colombo et al., found expression of Plp1, a Schwann cell precursor marker, in melanoblasts and melanocytes (Colombo et al., 2012). Similarly, single cell analysis of cochlear Plp1 positive embryonic ganglion cells found clusters expressing melanocyte markers (Tasdemir-Yilmaz et al., 2021). Finally, Kaucka et al. reported that nerve associated Schwan cell precursors contribute extracutaneous melanocytes to the heart, meninges and supraorbital locations amongst others (Kaucka et al., 2021).

Our data suggests that pigmented cells in the inner ear arise both through melanoblast precursors from neural crest migrating dorsolaterally under the dermis, and also from Schwann cell precursors associated with the cochleovestibular ganglion. As the future intermediate cells approach the cochlear duct at around E12.5 they express either markers for both glial cells and melanocytes, consistent with a Schwann cell precursor multipotent progenitor, or just melanocyte markers. At P6, most of the intermediate cells of the stria vascularis are labeled by Plp1-Cre suggesting their Schwann cell precursor origin. However, we still see intermediate cells that are Plp1 negative, which likely originated from melanoblast precursors.

### Postnatal Development of Intermediate Cells

Intermediate cells of the stria vascularis begin ingressing into the lateral wall of the cochlea in a basal to apical gradient starting at around E15.5 and continue to ingress until birth (Figure 2). By postnatal day 0, intermediate cells occupy their final position between marginal and basal cells. At this point, the cells have begun their differentiation into melanocytes but still retain makers of multipotent neural crest (Renauld et al., 2021). Between birth and postnatal day 6, intermediate cells begin to change morphology and go from a round shape to a more elongated shape that sends protrusions that interdigitate with the other cell types of the stria forming tight junctions (Cable et al., 1992; Tachibana, 1999). By the onset of hearing, around P15, intermediate cells have fully differentiated and adopted their mature shape. They occupy the whole width of the stria, from the spiral prominence to the start of Reisner’s membrane. Our data suggests that intermediate and basal cells proliferate during embryonic stages and continue to do so after birth. The proliferation declines rapidly after postnatal day 6 which can be related to the stria vascularis reaching its final volume. However, we cannot discard the possibilty that absence of labeling in posnatal stages maybe due to a decreased rate of cell proliferation in which case EdU might clear from the cells before incorporation. We did not detect significant cell death either embryonically or in the first 2 weeks after birth (Figure 3). Previous studies have shown that intermediate cells resume proliferation in adult mice (Conlee et al., 1994).

### Other Neural Crest Contributions to the Cochlea

Most lineage analyses have described glial cells of the VIIIth ganglion and pigmented cells of the cochlea and vestibular organs as the only neural crest derivatives in the inner ear (Mao et al., 2014; Sandell et al., 2014; Karpinski et al., 2016). A notable exception is controversial work by Freyer et al., that used not only a Wnt1-Cre driver but also a Pax3-Cre driver to label neural crest derived cells. This study showed Pax-3 labeled cells in the sensory organ of Corti (Freyer et al., 2011). A caveat of these studies is that Wnt1-Cre does not label all neural crest, and that Pax3 expression during neural tube closure is broader than just prospective neural crest, raising the possibility that other tissues might have contributed to labeling in the inner ear (reviewed in (Ritter and Martin, 2019). However, similar studies found no contribution of neural crest to the inner ear other than the glia of the VIIIth ganglion and melanocytes (Mao et al., 2014; Karpinski et al., 2016).

We have actually observed both phenotypes using the same mouse wnt1-Cre2 and Ai3 reporter lines in different studies. The present study, seems in agreement with Freyer results. We do observe EYPF positive cells present in the sensory epithelium. However, this expression does not reflect contribution of migratory neural crest. When we looked at premigratory stages (E8.5), we found expression of Wnt1-Cre2 extending beyond the dorsal neural tube and into the otic placode. We believe those positive cells originate from Wnt1 expression in the otic placode and not from neural crest cells migrating into the inner ear as shown in the Supplementary Figure S2A. In contrast, in a previous study when we did similar lineage tracing using Wnt1-CRE2 and reporter lines, we never observed neural crest contribution in the inner ear besides pigmented cells or glia of the VIIIth ganglion (Renauld et al., 2021).

The only difference between this study and our previous one, was the genetic background of the mice used. In our previous study, all mice had a congenic ICR background. Because here we wanted to see pigment in the melanocytes, we used a mixed background that included B6 mice in our breeding schemes. This raises the possibility that genetic background might affect Wnt1 expression. Such background differences in phenotypic expression have been previously described and it is known that genetic modifiers can alter gene expression outcomes in the ear (Kiernan et al., 2007; Kane et al., 2012).

## Conclusion

Here, we studied the development of intermediate cells of the stria vascularis from early migration of neural crest to differentiation in the postnatal cochlea. We showed that the pigmented cells that will form intermediate cells differentiate both from migratory neural crest that adopt a dorsolateral migration pathway and from nerve-associated Schwann cell precursors. Between E12.5 and E14.5 these cells line up above the roof of the cochlea on the site of the future lateral wall. Starting at E15.5, probably in response to a signal or signals from the cochlear epithelium, these cells incorporate into the stria vascularis following a basal to apical gradient reminiscent of the differentiation of the organ of Corti. Cells in the stria proliferate as the cochlea develops and this proliferation declines rapidly after postnatal day 6.

## Data Availability

The original contributions presented in the study are included in the article/Supplementary Material, further inquiries can be directed to the corresponding author.

## Supplementary Material

Supplementary Figure S1 Early migration of MITF+ neural crest cells MITF+ neural crest migrating both dorsally and ventrally towards the otic vesicle where they will become intermediate cells of the stria and melanoblasts of the vestiblar system, at E10.5 (MITF labeled in red, cell nucleus in blue). NT: neural tube, OV: otic vesicle.

Supplementary Figure S2 Wnt1-Cre2 labels the otic placode in a mixed mouse background **(A)** Lineage tracing using Wnt1-Cre2 mice crossed to an EYFP reporter line was visualized by staining with the anti-GFP antibody in green and DAPI for nucleus in blue at E8.5 and P0. NT: Neural tube. Dotted line delimited the neural tube and the otic placodes. Arrows: YFP positive cells; arrowhead: positive Deiters’ cell. Asterisk: positive inner hair cell. **(B)** percentage of Wnt1Cre-YFP positive cells in the organ of Corti counted on the whole mount. *n* = 5 from 2 different litters.
