# Supplementary figures and images for: Intermediate Cells of Dual Embryonic Origin Follow a Basal to Apical Gradient of Ingression Into the Lateral Wall of the Cochlea
Source: Front Cell Dev Biol. 2022 Mar 8;10:867153. doi: 10.3389/fcell.2022.867153 (PMC8964366; doi:10.3389/fcell.2022.867153)

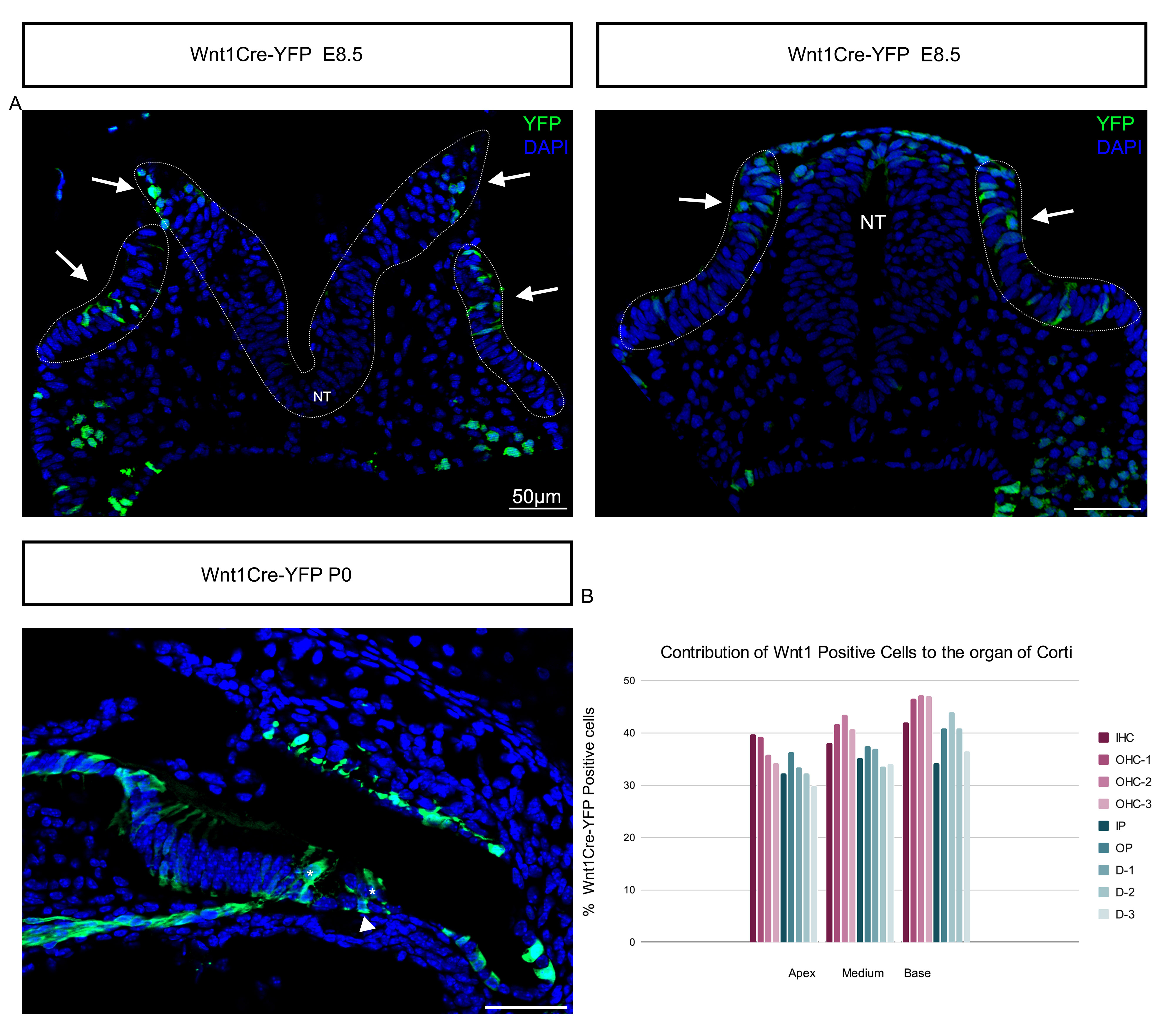

Supplement: Supplementary file 1 [file Image1.JPEG]

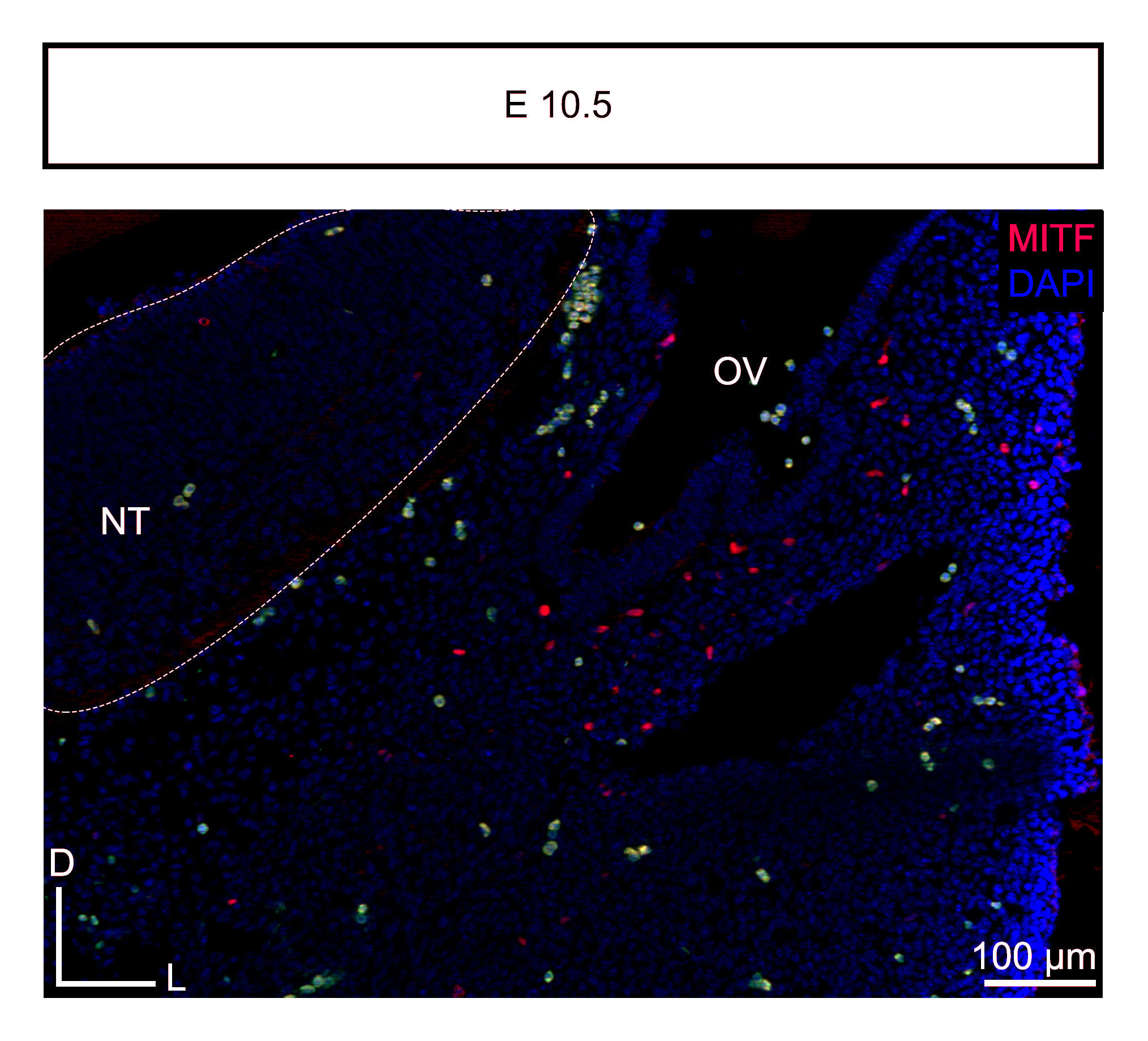

Supplement: Supplementary file 2 [file Image2.JPEG]
